# A Federated Recommendation System with a Dual-Layer Multi-Head Attention Network and Regularization Strategy

**DOI:** 10.3390/e27111112

**Published:** 2025-10-28

**Authors:** Qianxiao Yue, Xiangrong Tong

**Affiliations:** 1 School of Computer and Control Engineering, Yantai University, Yantai 264005, China; 2 Key Laboratory of Sea-Air Information Perception and Processing Technology of Shandong Provincial, Yantai 264000, China

**Keywords:** federated learning, recommender systems, multi-head attention, user–item interactions, regularization

## Abstract

Federated recommendation (FedRec) aims to provide effective recommendation services while preserving user privacy. However, in a federated setting, a single user cannot access other users’ interaction data. With limited local interactions, existing FedRec models struggle to fully exploit interaction information to learn users’ preferences. Moreover, training recommendation models in decentralized FedRec scenarios suffer from a risk of overfitting. To address the above issues, we propose a federated recommendation system with a dual-layer multi-head attention network and regularization strategy (FedDMR). First, FedDMR initializes clients’ local recommendation models. Subsequently, clients perform local training based on their private data. Our dual-layer multi-head attention network is designed to perform attention-weighted interactions on user and item embeddings, progressively capturing local interaction information and generating interaction-aware embeddings, thereby enriching users’ feature representations for modeling personalized preferences. Then, a regularization strategy is employed to guide updates to clients’ models by constraining their deviation from the global parameters, which effectively mitigates overfitting caused by limited local data and enhances the generalizability of the models. Finally, the server aggregates the clients’ uploaded parameters for this round. The entire training process is implemented through the federated learning framework. Experimental results on three datasets demonstrate that FedDMR achieves an average improvement of 2.63% in AUC and precision compared to the recent federated recommendation baselines.

## 1. Introduction

Traditional recommendation systems often require servers to centrally collect large amounts of raw user data, which typically contain sensitive user privacy information. However, uploading personal data to servers poses significant privacy and security risks to users [1]. Federated recommendation (FedRec) [2] achieves the goal of keeping raw data within the domain and making the data usable but invisible [3] by localizing user data and iteratively transmitting only model parameters between the server and clients to train a recommendation system.

Unlike centralized recommendation systems, individual users cannot access the interaction data of others for recommendation purposes in the federated setting, which confines the model to learning user preferences based exclusively on limited local interactions. Thus, FedRec is achieved by effectively modeling the features of users and items as well as their local interactions. Existing FedRec models [4,5,6,7,8,9] typically employ methods such as matrix factorization or simple neural networks [10] to process user and item features. They first map these high-dimensional features into a low-dimensional vector space to generate feature embeddings; these feature embeddings are then concatenated and used as the input to the recommendation model for prediction. Although these methods can learn simple interactions between feature embeddings, they struggle to deeply exploit interaction patterns and leverage them effectively for preference modeling.

Attention mechanisms [11] enable the model to selectively focus on the most relevant parts of the input data, and they are widely used in centralized recommendation systems to capture user–item interaction relationships. In the context of FedRec, this means that the recommendation model can weigh different feature embeddings’ interactions and model them better. While the mechanisms in federated sequential recommendation [12,13,14,15,16] are often used to model the temporal evolution of user interests, they are rarely applied in collaborative filtering FedRec scenarios considered in this paper. To address this gap, we innovatively introduce multi-head attention into our work to focus on multiple aspects of user preferences.

In addition, insufficient data on clients may lead to overfitting during the training of recommendation models. Then, the parameters uploaded by clients tend to convey user-specific biases rather than generalizable patterns. This not only degrades the overall recommendation performance but also reduces the effectiveness of collaborative knowledge-sharing across clients. The existing methods to address model overfitting mainly fall into three categories: regularization [17], dropout [18], and data augmentation [19]. Dropout may disrupt the stability of model parameters, thereby affecting the convergence of the federated recommendation model. Data augmentation requires the transmission of more data across clients, which increases communication costs. Thus, regularization has become a crucial method of balancing personalization and generalization by constraining the direction and magnitude of parameter updates.

To this end, we propose a federated recommendation system with a dual-layer multi-head attention network and regularization strategy (FedDMR). The dual-layer multi-head attention network employs progressive attention modeling to fully exploit interaction information, and the regularization strategy effectively prevents overfitting by imposing global constraints on the shared parameters trained across clients. The combination of these two components enables more accurate modeling of user-specific preferences while ensuring the generalization capability of the model.

Our dual-layer multi-head attention network is designed with an interactive attention layer and an aggregative attention layer. While the interactive attention layer captures direct interactions between users and items, the aggregative attention layer integrates higher-order interaction information and generates embeddings for these interactions. This provides richer interactive feature representations for modeling users’ preferences. Then, the regularization strategy is employed during local training with two regularizers, namely the discrepancy regularizer and the L1 regularizer. The discrepancy regularizer imposes global constraints on the updates of clients’ public parameters, and then the L1 regularizer encourages the sparsity of the parameters, thereby discarding some unimportant information. This regularization strategy can effectively enhance the model’s generalizability. Moreover, the application of a pre-trained model for federated recommendation has achieved great success [9,20,21]. Inherent knowledge can be leveraged using the parameters of the pre-trained model to initialize the federated recommendation model, enabling the federated recommendation system to be optimized from a better starting point and accelerating its convergence. Our method follows this approach and also achieves promising results.

We now discuss how our FedDMR differs from some representative FedRec methods [5,6,8,9]. Existing methods mainly follow the neural collaborative filtering paradigm [5], relying on MLP-based and shallow concatenation operations for interaction modeling, which cannot fully capture complex user–item dependencies. PFedRec [6] and FedRAP [8] introduce dual personalization and additive personalization, respectively, but still lack the explicit modeling of interaction patterns. FedPA [9] introduces low-rank adapters to achieve parameter-efficient personalization, but it provides only shallow semantic representations and fails to capture interaction structures. Our FedDMR progressively extracts both direct and high-order interaction representations through a dual-layer multi-head attention network, enabling a more expressive representation of user preferences under data-sparse federated settings. Furthermore, FedDMR integrates attention-based feature modeling with regularization-based optimization, achieving a balanced trade-off between personalization and generalization. This cooperative design enables FedDMR to outperform prior FedRec methods.

In this study, we evaluate the performance of FedDMR on three benchmark datasets; the experimental results demonstrate that FedDMR outperforms several state-of-the-art baselines. Furthermore, we validate the model’s scalability in real-world applications. By compressing computationally intensive dual-layer multi-head attention networks into a lightweight net, we address the computational challenges of deploying the attention networks on edge devices. The main contributions of this work are summarized as follows:

- We design a dual-layer multi-head attention network in the local recommendation model. The multi-head attention network enables models to focus on different aspects of user interests and capture preference patterns across various item features, thereby providing a more comprehensive understanding of users’ diverse interests.
- We introduce a regularization strategy to guide the updates of local models by incorporating two regularizers, which steer the updates of local models towards the global model, effectively preventing the risk of overfitting.
- Extensive experiments on three benchmark datasets demonstrate the outstanding performance of FedDMR against baselines. Additionally, FedDMR also shows excellent scalability when deployed to clients with limited computational capability.

This paper is organized as follows. Section 2 reviews the related work. Section 3 defines the task of federated recommendation and describes the basic recommendation model used in our framework. Section 4 introduces our proposed FedDMR and the overall training algorithm in detail. Section 5 discusses the privacy-preserving and model scalability of FedDMR. Section 6 evaluates the performance of FedDMR. Finally, Section 7 concludes this paper and discusses future work.

## 2. Related Work

### 2.1. Federated Recommendation Systems

Federated recommendation systems have become relatively mature as a privacy-preserving paradigm that enables recommendation model training without exposing users’ raw data. Early studies such as FCF [4] and FedFast [22] adopt classical collaborative filtering or matrix factorization under federated settings, allowing clients to update local parameters while the server aggregates shared gradients. MetaMF [7] introduces a meta-learning matrix factorization framework for FedRec. Subsequent works have introduced neural network architectures to enhance representation learning. FedNCF [5] extends neural collaborative filtering (NCF) to the federated setting, where users train local NCF models on private interaction data and share only model updates with the server. PFedNCF [23] builds upon FedNCF by incorporating personalized model components, allowing each client to fine-tune shared NCF representations with local adaptations. FedGNN [24] applies graph neural networks to the FedRec scenario, where each client learns user–item interaction graphs locally and shares encrypted model parameters with the server. FedRecon [25] proposes a personalized federated learning method that separates global and local representations, where each client locally reconstructs personalized parameters while sharing only global components. PFedRec [6] introduces a dual personalized FedRec framework, which captures user preferences using a personalized score function and fine-grained personalization on item embeddings that learns both global and user-specific representations. FedPA [9] incorporates low-rank adapter layers into local neural recommendation models, performing personalized modeling through low-rank matrix decomposition of user features.

Recently, several advanced federated recommendation methods have emerged to align with new research directions. ClusterFedMet [26] improves personalization by capturing user group similarities and rapid adaptation across clients. FedDAE [27] extends FedRec into a denoising autoencoder framework, where each client trains local autoencoders to learn user–item interaction representations and shares encoded parameters for global aggregation. IFedRec [28] separates item attributes from user interactions and simultaneously learns two types of item representations on both the server and client sides. Moreover, privacy-preserving research has explored certified unlearning mechanisms [29], which enables users to remove their contribution from the federated model without retraining. In addition, FedCIA [30] introduces a new aggregation paradigm for federated recommendation that focuses on sharing collaborative information rather than model parameters.

However, most existing FedRec methods rely on matrix factorization or single-layer MLP for interaction modeling, which restricts their ability to capture hierarchical and high-order user–item interaction patterns. This results in these methods still having limited interpretability and expressiveness in modeling user preferences. Therefore, it is necessary for FedRec methods to better model the interactions between users and items.

### 2.2. Attention-Based Recommendation Models

Attention mechanisms have been widely applied in recommendation systems to model complex user–item interactions and capture user preferences. DIN [31] introduces an attention-based idea to recommendation systems, which dynamically assigns different weights to users’ historical behaviors according to the relevance of the target item, enabling personalized interest representation. SASRec [32] employs self-attention to model sequential user–item interactions, effectively learning long-term dependencies without relying on recurrent networks. BERT4Rec [33] applies the bidirectional self-attention architecture of BERT to sequential recommendation. It models user–item interaction sequences in both forward and backward directions, enabling the capture of comprehensive contextual dependencies among items. SNR [34] employs a multi-head attention-based framework to jointly capture users’ multi-factor and multi-faceted preferences in sequential recommendation. UPRec [35] employs a hierarchical multi-head attention network to jointly model users’ short-term and long-term preferences in sequential recommendation.

However, most existing attention-based recommendation methods are designed for centralized settings and require access to all users’ interaction histories, which makes them unsuitable for federated scenarios. Recently, several studies have explored federated sequential recommendation, which extends Transformer- or RNN-based sequence models to federated environments. FedKAT [12] employs an attention-based Transformer architecture to model users’ sequential behaviors under the FedRec framework. FedEM [15] utilizes masked self-attention networks to model users’ sequential behaviors and capture temporal dependencies within their interaction histories. MRFF [16] leverages attention-based foundation models to capture users’ multifaceted preferences under federated settings.

These methods primarily focus on temporal behavior modeling, capturing the order and dynamics of user interactions through sequence encoders. However, they are not applicable to the collaborative filtering scenarios in this paper, where user–item interactions are represented as static or implicit feedback matrices rather than sequential logs. To address this issue, our FedDMR introduces a dual-layer multi-head attention network into the federated collaborative filtering recommendation to better capture user–item interactions without assuming sequential order. This attention network can hierarchically model user–item interactions from both direct and high-order perspectives, thereby providing richer interactive feature representations to capture users’ preferences.

### 2.3. Regularization in Federated Learning

Regularization has been widely explored in federated learning to address data heterogeneity and prevent models from being prone to local overfitting or global divergence [36]. FedProx [37] restricts the deviation of local parameters from global parameters by adding an L2 regularizer to the local loss function, forcing the optimization direction of clients to approach the global model. FedDyn [38] introduces a dynamic regularizer to correct the optimization direction of clients through gradient alignment. FedReg [39] reduces knowledge forgetting by regularizing local training parameters with generated pseudo data. FedRecon [25] adapts regularization for personalized fine-tuning on user-level data.

Although these regularization methods are designed for generic federated learning, they have not been tailored to the context of FedRec. But, their core idea of using regularization to narrow the gap between local and global parameters still inspired us. Our FedDMR introduces a regularization strategy that incorporates both a discrepancy regularizer and an L1 regularizer to impose global constraints on the public-shared parameters of the local models, thereby effectively mitigating the risk of overfitting. The private parameters of the users are kept locally for personalized modeling and are not subject to regularization constraints. This regularization strategy can be effectively integrated into FedRec scenarios and enhance the model’s generalizability.

## 3. Preliminaries

### 3.1. Task Definition

Our work utilizes a pre-trained model to serve the federated recommendation system. Therefore, we split each dataset into two subsets: one for pre-training and the other for federated training. For each dataset, we first divide all users into two subsets based on a certain user attribute, such as user active degree (which is used in our experiments). For user attributes, there are generally several values that can be used for classification. These values can be divided into two categories: one used to identify feature-group users (e.g., users with a higher active degree), and the other used to identify non-feature-group users. Then, we randomly select 80% of the feature-group users and 20% of the non-feature-group users to form the open users that will be used to pre-train the model. The remaining users are kept as private users for federated training. Then, we partition the corresponding interaction records of open users and private users into two sets, $D^{pre}$ and $D^{fed}$, to separately train the pre-trained model and the federated recommendation system.

For the pre-trained model, we use the centralized training approach of recommender systems on the public dataset $D^{pre}$. After pre-training, we use the parameters saved from the pre-trained model to initialize the federated recommendation system. In dataset $D^{fed}$ for federated training, there are *n* users and *m* items; each user *i* possesses a private set of interaction records $D^{fed}\left(i\right)$. These users aim to collaboratively build a recommendation system based on locally trained models.

### 3.2. Basic Recommendation Model

Our recommendation model consists of three basic parts: a user embedding module, an item embedding module, and a prediction function. We first input the attributes of users and items to obtain user embedding vectors and item embedding vectors. Then, these embedding vectors are concatenated and fed into the prediction function to generate predicted ratings. The users’ interaction records serve as auxiliary information to guide the updates. We assume that the users’ interaction records are implicit feedback, where the label is $r_{ij}=1$ if user *i* has interacted with item *j* and $r_{ij}=0$ otherwise.

User Embedding Module: Each user has multiple attributes. For each attribute *k*, there is an embedding table $E_{k}\in R^{p\times d}$, where *p* is the total number of unique attribute values of attribute *k* and *d* is the embedding dimension. Then, for each user *i* with attributes $A_{i}$, we obtain the embedding vectors from all attribute embedding tables based on attribute values and obtain the user embedding by concatenating them as follows:

(1) $$u_{emb}^{i}=Concat\left(E_{u}{\left(A_{i}^{k}\right)}_{k=1}^{\left|A_{i}\right|}\right),$$

where $\left|A_{i}\right|$ is the total attribute number of user *i*.

Item Embedding Module: Similar to user embeddings, for item *j* with attributes $\left|A_{j}\right|$, we obtain the item embedding tables and the item embedding:

(2) $$v_{emb}^{j}=Concat\left(E_{v}{\left(A_{j}^{k}\right)}_{k=1}^{\left|A_{j}\right|}\right),$$

where $\left|A_{j}\right|$ is the total attribute number of item *j*.

Prediction Function: Then, the MLP (multilayer perceptron) is used as the prediction function, and the predicted ratings output by the prediction function can be formalized as follows:

(3) $${\hat{r}}_{ij}=MLP\left(Concat\left(u_{emb}^{i},v_{emb}^{j}\right)\right),$$

where ${\hat{r}}_{ij}$ is the predicted rating for user *i* on item *j*.

Recommendation Loss: To update the local recommendation model, we use binary cross-entropy as the loss function, and the recommendation loss is as follows:

(4) $$L_{rec}\left(\theta_{rec}^{i}\right)=-\sum_{\left(i,j\right)\in D^{fed}\left(i\right)}r_{ij}log{\hat{r}}_{ij}+\left(1-r_{ij}\right)\log\left(1-{\hat{r}}_{ij}\right),$$

where $\theta_{rec}^{i}$ are the basic recommendation model parameters of user *i*, including user embedding tables $\theta_{ue}$, item embedding tables $\theta_{ve}$, and MLP parameters $\theta_{mlp}^{i}$.

The recommendation model used for pre-training comprises only the aforementioned basic components; its training parameters $\theta_{rec}^{pre}$ are subsequently employed to initialize the users’ parameters $\theta_{rec}^{i}$ during federated training. For FedRec models, FedPA [9] incorporates low-rank adapters and an adaptive gating mechanism into the prediction function; here, we retain these modules in our models. The item embedding table parameters $\theta_{ve}$ are derived from modeling the basic attributes of items. Since all items are shared between public and private users, their fundamental features have already been learned during pre-training, and then we freeze the updates of $\theta_{ve}$ during federated training. The user embedding table parameters $\theta_{ue}$ and the prediction function parameters $\theta_{mlp}$ are user-specific parameters and are updated normally during training.

## 4. FedDMR

**Figure 1:**
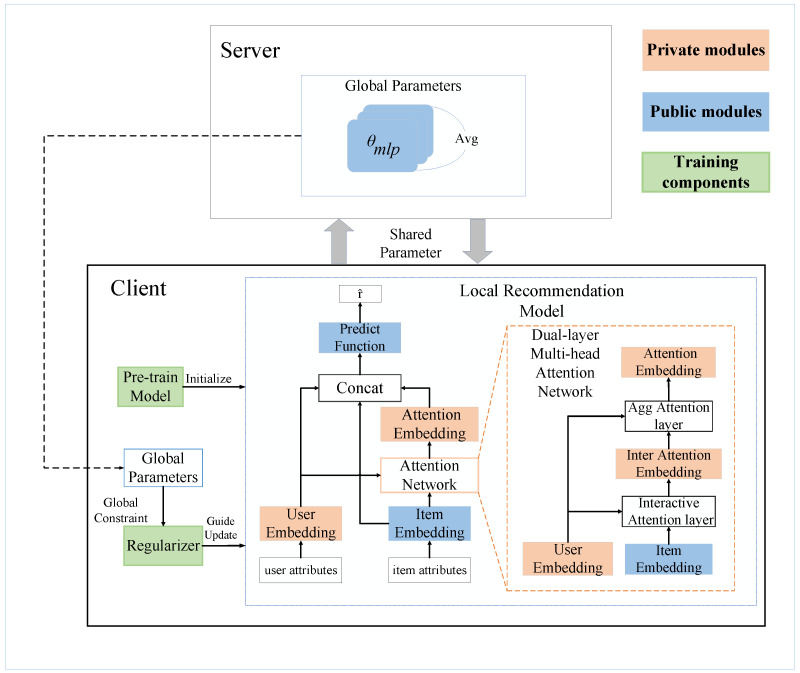
The framework of FedDMR. Our system adopts a client–server architecture. Clients are responsible for training and updating local models. The server is responsible for aggregating updated public parameters.

In this section, we propose a federated recommendation system with a dual-layer multi-head attention network and regularization strategy (FedDMR). The model architecture is illustrated in Figure 1. Our system adopts a client–server architecture. First, we load the parameters of the pre-trained model to initialize the local recommendation models. Subsequently, clients conduct local training using their private data. During this process, the dual-layer multi-head attention network is used to exploit the interaction information between user embeddings and item embeddings and then generate interaction embeddings for prediction. Meanwhile, the updates of the clients’ parameters are guided by the regularizers, which exert their effect by imposing global constraints on local models. After local training, the updated publicly shared parameters of client *i* (denoted as $\theta_{public}^{i}$, including the prediction function parameters $\theta_{mlp}^{i}$) are uploaded to the server for global aggregation. Subsequently, the server distributes the aggregated global model parameters to clients for the next training round. This process iterates continuously until the model converges. The main notations used in this paper are summarized in Table 1.

In this section, we first introduce the dual-layer multi-head attention network and the regularization strategy. Subsequently, we summarize the workflow of the FedDMR in an optimization algorithm.

### 4.1. Dual-Layer Multi-Head Attention Network

In the federated collaborative filtering-based recommendation scenario [40], individual users can only access their own interaction data, so it is necessary for recommendation models to deeply capture the interactions between user and item embeddings for preference modeling. Existing recommendation methods (such as matrix factorization or simple neural networks) typically perform vector concatenation or dot-product operations on user embeddings and item embeddings. While these methods can learn simple interaction patterns between feature embeddings, they lack the ability to dynamically adjust the importance of interactions and fail to fully exploit deeper interactive information. To overcome this limitation, we introduce multi-head attention into the recommendation model, which can effectively model the complex interaction patterns between feature embeddings by dynamically assigning attention weights.

The motivation for adopting a dual-layer attention structure rather than a single-layer or conventional attention design lies in the nature of federated collaborative filtering: each client only has sparse and limited local interactions, making it difficult to capture high-order dependency signals between user–item pairs. While the first attention layer focuses on immediate pairwise correlations between users and items, the second attention layer can further integrate these fine-grained relations into higher-order semantic representations. This hierarchical design enables models to progressively refine interaction representations. Therefore, we design a dual-layer multi-head attention network that maximally excavates and exploits interactive information.

Interactive Attention Layer: This layer calculates interactive weights between user embeddings and item embeddings through multi-head attention and generates interactive attention embeddings. For user *i* on item *j*, the output can be expressed as follows:

(5) $$int_{emb}^{ij}=Multihead\left(u_{emb}^{i},v_{emb}^{j},v_{emb}^{j}\right),$$

where $u_{emb}$ is user embedding as a query, $v_{emb}$ is item embedding as a key and value, and $int_{emb}$ is interactive attention embedding. The core objective of a recommendation system is to recommend items that align with users’ interests, so we utilize user embeddings as the query rather than item embeddings. User embeddings directly reflect the characteristics and preferences of users; using user embeddings as queries can better capture users’ personalized needs. Using item embeddings as the key and value, the model can capture the similarities and differences between items, thus better understanding the relationships between items. Multihead represents the multi-head attention mechanism, and its internal implementation is as follows:

(6) $$Multihead\left(u,v,v\right)=Concat\left(head_{1},head_{2},\ldots ,head_{H}\right)W^{o},$$

(7) $$head_{h}=Attention\left(uW_{i}^{Q},vW_{i}^{K},vW_{i}^{V}\right),$$

where $W_{h}^{Q}$, $W_{h}^{K}$, and $W_{h}^{V}$ are the weight matrices for the *h*-th attention head, $W^{o}$ is the output weight matrix, and *H* is the number of attention heads. In our work, we configure the attention network with four attention heads by default, since we selected four attributes for users and items, respectively, from each dataset to model their embeddings. Aligning the number of attention heads with the number of attributes allows each head to specialize in capturing the interaction signals corresponding to a single attribute in the latent space, reducing interference among different attributes while avoiding the redundancy and noise introduced by an excessive number of attention heads.

Aggregative Attention Layer: While the interactive attention layer captures direct interactions between user embeddings and item embeddings, the aggregative attention layer further exploits these basic interactions by calculating the interactive weights between user embeddings and interactive attention embeddings. The output can be expressed as follows:

(8) $$att_{emb}^{ij}=Multihead\left(u_{emb}^{i},int_{emb}^{ij},int_{emb}^{ij}\right),$$

where $u_{emb}$ is user embedding as a query, $int_{emb}$ is interactive attention embedding as a key and value, $att_{emb}$ is aggregative attention embedding, and Multihead is defined the same as in the interactive attention module.

We originally intended to replace the item embeddings with aggregative attention embeddings for concatenation with user embeddings, then input them into the prediction function. However, this led to a degradation in the model’s performance because aggregated attention embeddings may overlook some important item feature information. So, we concatenate aggregative attention embeddings with user embeddings and item embeddings and use the concatenation as the input to the prediction function, then re-formulate the predicted ratings output by the prediction function in (3) as follows:

(9) $${\hat{r}}_{ij}=MLP\left(Concat\left(u_{emb}^{i},v_{emb}^{j},att_{emb}^{ij}\right)\right).$$

By inputting the aggregative attention embeddings into the prediction function as well, the model can gain a more comprehensive understanding of the input, thereby enhancing its predictive performance.

### 4.2. Regularization Strategy

In the distributed environment of federated learning, updates of the global model rely on updates of local models from all clients. However, the amount of data available on each client is usually limited [41]. This makes the model prone to overfitting local data, and the parameters uploaded by clients may convey user-specific biases, resulting in poor recommendation performance across all clients.

To mitigate the overfitting issue, we propose a regularization strategy to ensure that the updates of the local models are constrained by the global model. This strategy introduces two regularizers to optimize the local loss function: the discrepancy regularizer and the L1 regularizer. The discrepancy regularizer constrains the deviation between local and global model parameters, encouraging consistency among clients while still allowing personalization, and the L1 regularizer plays a supplementary role by applying sparsity control to model parameters. The combination of the two regularizers enables the model to achieve stable convergence and robust personalized recommendations under federated settings.

Specifically, for each client participating in federated training, we first use the discrepancy regularizer to calculate the L2-norm difference between its model’s public parameters and the global parameters. The mathematical expression is as follows:

(10) $$L_{\mathrm{diff}\;}=\lambda \cdot \sum_{\theta \in \Theta_{\mathrm{public}\;}}{∥\theta_{\mathrm{public}\;}^{i}-\theta_{\mathrm{global}\;}∥}_{2}^{2},$$

where $\Theta_{\mathrm{public}\;}$ is a set of public parameters, $\theta_{\mathrm{public}\;}^{i}$ are the public parameters for client *i*, $\theta_{\mathrm{global}\;}$ are the global parameters, and λ is the hyperparameter that controls the strength of discrepancy regularization.

Then, the L1 regularizer encourages the sparsity of the public parameters by calculating the L1-norm of the model parameters, thereby reducing parameter complexity. The mathematical expression is as follows:

(11) $$L_{L1}=\mu \cdot \sum_{\theta \in \Theta_{\mathrm{public}\;}}{∥\theta_{\mathrm{public}\;}^{i}∥}_{1},$$

where μ is the hyperparameter that controls the strength of L1 regularization. The model needs to quickly learn the basic features of the data during early training; using a smaller regularization strength at this stage can help the model converge faster. As training progresses, gradually increasing the regularization constraints can effectively prevent the model from overfitting to the local training data, thus improving the model’s performance on the global data and test data.

During the training process, regularizers are added to the loss function and directly influence the updates of the parameter. This allows for real-time adjustment of the direction and magnitude of the updates. By summing the recommendation loss and the regularizer, we obtain the total loss for client *i* as follows:

(12) $$L_{i}=L_{rec}+L_{diff}+L_{L1}.$$

By incorporating the regularizer into the loss function, the gradient calculation takes into account not only the loss of local data but also the constraints of the global model, thereby reducing the risk of overfitting. Moreover, the model focuses more on the general patterns of the data rather than local features during training, then enhances the model’s generalizability.

### 4.3. Objective Function

FedDMR aims to build a personalized federated recommender system that delivers effective recommendations to each user. The objective function of the federated recommendation system can be formalized as follows:

(13) $$\min_{{\left\{\theta_{mod}^{i}\right\}}_{i=1}^{n}}\sum_{i=1}^{n}\frac{a_{i}}{a}L_{i}\left(\theta_{mod}^{i}\right),$$

where $L_{i}\left(\theta_{mod}^{i}\right)$ is the local loss of client *i*, $\theta_{mod}^{i}$ are the model parameters of client *i*, including basic recommendation model parameters $\theta_{rec}^{i}$ and attention network parameters $\theta_{att}^{i}$, $a_{i}$ is the amount of data on client *i*, and *a* is the total amount of data across all clients. Here, we employ a weighted aggregation approach to optimize federated system parameters.

### 4.4. Algorithm

In this subsection, we will provide a detailed description of the entire training process of FedDMR in Algorithm 1.
**Algorithm 1** The training process of FedDMR.**Input:** Public dataset $D^{pre}$, Private dataset $D^{fed}\left(i\right)$, number of clients *n*, learning rate η, number of rounds $t_{1}$, number of local iterations $t_{2}$ **Pre-train:** Train a recommendation model using open dataset $D^{pre}$ and save its model parameters $\theta_{ue}^{pre}$, $\theta_{ve}^{pre}$, $\theta_{mlp}^{pre}$ **Global Procedure:**
  1:**for** each client index i=1,2,…,n in parallel **do**  2:     Initialize $\theta_{ue}$, $\theta_{ve}$, $\theta_{mlp}$ with $\theta_{ue}^{pre}$, $\theta_{ve}^{pre}$, $\theta_{mlp}^{pre}$  3:**end for**  4:**for** $a=1,2,\ldots ,t_{1}$ **do**  5:     $S_{a}\leftarrow$ randomly select $n_{s}$ clients from *n* clients  6:     **for** each client index $i\in S_{a}$ in parallel **do**  7:         $\theta_{mlp}^{i},{\hat{r}}_{i}\leftarrow \mathrm{ClientUpdate}\left(D^{fed}\left(i\right),\theta_{ue},\theta_{ve},\theta_{mlp}\right)$  8:         Upload $\theta_{mlp}^{i}$ to the server  9:     **end for**10:     $\theta_{mlp}\leftarrow \frac{1}{n_{s}}\sum_{i=1}^{n_{s}}\theta_{mlp}^{i}$                       ▹ Server Aggregation11:**end for**12:**Return:** Predicted ratings $\hat{R}={\left[{\hat{r}}_{1},{\hat{r}}_{2},\ldots ,{\hat{r}}_{n}\right]}^{T}$ **Client Update:**
  1:**for** $b=1,2,\ldots ,t_{2}$ **do**  2:     $att_{emb}^{i}\leftarrow \mathrm{MultiheadAttention}\left(u_{emb}^{i},v_{emb}\right)$  3:     ${\hat{r}}_{i}=MLP\left(\langle u_{emb}^{i},v_{emb},att_{emb}^{i}\rangle \right)$  4:     Compute partial gradients $\nabla_{\theta_{ue}}L$, $\nabla_{\theta_{mlp}^{i}}L$, $\nabla_{\theta_{att}^{i}}L$ with (13)  5:     Update $\theta_{ue}\leftarrow \theta_{ue}-\eta \nabla_{\theta_{ue}}L$  6:     Update $\theta_{mlp}^{i}\leftarrow \theta_{mlp}^{i}-\eta \nabla_{\theta_{mlp}^{i}}L$  7:     Update $\theta_{att}^{i}\leftarrow \theta_{att}^{i}-\eta \nabla_{\theta_{att}^{i}}L$  8:**end for**  9:**Return:**$\theta_{mlp}^{i},{\hat{r}}_{i}$


First, we pre-train a recommendation model on the server using a public dataset $D^{pre}$. After pre-training, the server stores the parameters of the pre-trained model, including user embedding tables $\theta_{ue}^{pre}$, item embedding tables $\theta_{ve}^{pre}$, and prediction function parameters $\theta_{mlp}^{pre}$.

In federated recommendation systems, each user *i* has a private interaction dataset $D^{fed}\left(i\right)$. Initially, each client receives the parameters of the pre-trained model from the server to initialize the user embedding tables $\theta_{ue}$, item embedding tables $\theta_{ve}$, and prediction function parameters $\theta_{mlp}^{i}$$\left(i=1,2,\ldots ,n\right)$ of the local recommendation model. We freeze the updates of $\theta_{ve}$ during federated training. In each training round, the server randomly selects a subset of clients (denoted as $S_{a}$) to participate in the training. During the local training of user *i*, the dual-layer multi-head attention network captures the interactions between user embeddings $u_{emb}^{i}$ and all item embeddings $v_{emb}$ that user *i* has interacted with, then generates aggregated attention embeddings $att_{emb}^{i}$ of user *i*. We input $u_{emb}^{i}$, $v_{emb}$, and $att_{emb}^{i}$ into the prediction function together to provide a richer feature representation for prediction. Meanwhile, we employ the regularization strategy to guide updates to local models, ensuring that these updates are constrained by the global model.

After a round of training, the clients retain $\theta_{ue}$ locally and transmit $\theta_{mlp}^{i}$ to the server for global aggregation. Then, in the next round, the server distributes the aggregated prediction function parameters ${\left\{\theta_{mlp}^{i}\right\}}_{i=1}^{N}$ to the clients for continued training. While training is completed, FedDMR outputs the predicted ratings $\hat{R}$ to guide the recommendation.

## 5. Discussions

### 5.1. Discussion of Privacy-Preserving and Communication Costs

The distributed training framework of federated learning can embody data locality and avoid direct exposure to private user data. In this paper, FedDMR retains private model parameters locally and freezes the updates of item embedding tables that are publicly shared, thereby avoiding their upload to the server. By keeping the parameters of user embedding tables and attention networks locally, the server has access to fewer model parameters, which reduces the risk of inferring users’ private data. By reducing the amount of public parameters uploaded, communication costs between the server and users are significantly reduced, which is particularly beneficial for recommender systems with extensive user data.

Furthermore, we integrate the local differential privacy [42] technique to add Laplacian noise to the publicly shared parameters before uploading them to the server. Compared with other privacy-preserving techniques such as DP-FedAvg (which injects noise into gradients and introduces higher performance loss) and secure aggregation (which encrypts model updates and entails higher communication and computation overheads), LDP provides a lightweight and effective solution for FedRec. It requires no cryptographic computation and can be applied locally before communication, thereby maintaining low communication costs while being lossless in accuracy. For FedDMR with LDP, as the noise intensity increases (i.e., stronger privacy protection), the perturbation added to public parameters becomes larger, leading to an inevitable degradation in model accuracy. Moderate noise levels achieve an appropriate balance between privacy and accuracy, ensuring that the model remains effective while preserving user data confidentiality. The detailed experimental analysis can be found in Section 6.9.

### 5.2. Discussion of Efficiency and Model Scalability

Dual-layer multi-head attention networks are computationally intensive. Deploying attention networks directly to clients in a federated setting poses challenges in terms of limited storage and computational capacity. To address this issue, we leverage the Linformer [43] technique, which introduces low-rank matrices to compress the dimensions of matrices *K* and *V*, then the computational cost of the attention networks can be significantly reduced. The detailed analysis is as follows.

For each user *u*, there is an interaction sequence *L*; that is, the number of items the user has interacted with. In our recommendation model, given the embedding dimension *d* and an *a*-Layer MLP, the computational complexity of training the basic recommendation model without attention networks is $O\left({\mathrm{aLd}}^{2}\right)\approx O\left({\mathrm{Ld}}^{2}\right)$. In the computation of an attention network, there are matrices $Q,K,V\in R^{L\times d}$; the computational complexity mainly comes from the calculation of $QK^{T}\in R^{L\times L}$ and the subsequent multiplication with V, which can be approximated as $O\left(L^{2}d\right)$. While incorporating dual-layer attention networks, the computational complexity turns into $O\left(2\left({\mathrm{Ld}}^{2}+L^{2}d\right)+{\mathrm{aLd}}^{2}\right)$. In real recommendation scenarios, users will interact with a large number of items, and the computational complexity can be approximated as $O\left(L^{2}d\right)$ since L≫d>a, which indeed increases computational costs. To reduce costs, we use low-rank matrices $E_{K},E_{V}\in R^{k\times L}$ to perform linear projections on K and V, respectively, in order to compress the sequence length to k≪L. Then, the computation of attention is redefined as $Q{\left(E_{K}K\right)}^{T}\in R^{L\times k}$ and the subsequent multiplication with $E_{V}V$, and the computational complexity of attention is reduced to $O\left(\mathrm{Lkd}\right)$. Since k≤d≪L, the computation complexity of training the models is reduced to $O\left(2\left({\mathrm{Ld}}^{2}+\mathrm{Lkd}\right)+{\mathrm{aLd}}^{2}\right)\approx O\left({\mathrm{Ld}}^{2}\right)$. This approach increases limited computational costs while improving model performance, effectively enhancing the scalability of the proposed dual-layer multi-head attention networks in real-world applications. The experimental validation can be found in Section 6.7.

## 6. Experiment

### 6.1. Experimental Setup

We evaluate FedDMR on three recommendation datasets: KuaiRand-Pure [44] and KuaiSAR [45] (KuaiSAR-S and KuaiSAR-R). The basic statistics of the datasets are shown in Table 2. We first split each dataset into a public dataset $D^{pre}$ and a private dataset $D^{fed}$. Specifically, for the KuaiRand-Pure dataset, we distinguish between feature-group and non-feature-group users based on the full_active attribute value of the user_active_degree attribute. Then, according to the dataset splitting logic in Section 3.1, a total of 16,232 users are designated as open users, while the remaining 11,053 users are designated as private users. For the KuaiSAR dataset, we distinguish between feature-group and non-feature-group users based on the 2, 5, 7 attribute values of the onehot_feat1 attribute. According to the dataset splitting logic, a total of 15,004 users are designated as open users, and the remaining 10,873 users are designated as private users. Then, the corresponding interaction records of the open users and the private users form the public set $D^{pre}$ for pre-training and the private set $D^{fed}$ for federated training, respectively. The private dataset for training the federated recommendation system is further split into training, validation, and test sets for each user based on timestamps, with a ratio of 6:2:2.

We evaluate model performance by calculating two evaluation metrics: AUC (area under curve) and precision. Typically, a higher AUC indicates a stronger classification ability of the model, while a higher precision suggests greater accuracy of the model’s predictions. All experimental results are repeated 10 times, and all results are in units of $1\times 10^{-2}$.

### 6.2. Baselines and Implementation Details

**Baselines**: To assess the effectiveness of FedDMR, we select five representative baselines, including FedNCF [5], PFedNCF [23], FedRecon [25], PFedRec [6], and FedPA [9]. Furthermore, we remove the pre-trained model from FedDMR to assess the contribution of the regularization strategy and the dual-layer multi-head attention mechanism for the federated recommendation system.

**Implementation Details**: All training and inference are conducted using PyTorch 2.0.1 (compiled with CUDA 11.7) on an GTX 1650 GPU and Intel Core i5-10200H CPU. For the hyperparameter settings in the experiment, we set the maximum number of global communication rounds and the maximum number of local training iterations on clients to 100 and 10, respectively. To ensure the fairness of the comparative experiments, all baseline models use a fixed latent embedding dimension of 8 and a batch size of 1024, with a learning rate of 0.1 and 4 user/item attributes.

For FedDMR, the prediction function adopts two hidden MLP layers whose architecture is 96 → 32 → 8, and then a linear output layer with a size of 8 → 1 is used to generate the predicted ratings. The input dimension of the first MLP layer is calculated as the product of the embedding dimension, the number of user/item attributes, and the number of embedding representations. The first MLP layer of other baseline models has a hidden layer size of 64 → 32, as it only uses embedding representations of users and items, whereas our FedDMR additionally incorporates attention embedding representations. The hidden layers use the ReLU activation function, while the output layer employs the Sigmoid activation function. The Adam optimizer is employed for all methods with weight decay of 1 × 10^−5^. For the pre-trained model, we use the same recommendation backbone described in Section 3.2, using the centralized training approach of recommender systems on the public dataset $D^{pre}$ for 50 epochs. The pre-trained parameters are then used to initialize the corresponding modules in the federated stage. For the experimental parameter settings of FedDMR, the dual-layer multi-head attention network is configured with 4 attention heads by default, and the parameters λ and μ that control the regularization strength are in the range of $\left\{10^{i}|i=-3,-2,-1,0,1,2\right\}$, and its default setting is 0.1. The detailed hyperparameter settings are shown in Table 3.

### 6.3. Comparison Analysis

Table 4 shows the AUC and precision metrics of our method and the comparison baselines on three datasets. We discuss the notable observations derived from the experimental results as follows:

- FedDMR generally performs well across all settings and datasets. It shows notable performance in AUC and precision under both “w/Warm” and “w/o Warm” settings. Its AUC achieves the best results on all datasets, with an average improvement of 3.01% compared to the second-best methods, and the highest improvement is 4.46% on the KuaiSAR-R dataset. Its precision does not achieve the best on the KuaiSAR-S dataset, but it shows an average improvement of 2.25% compared to the second-best methods in other cases.
- The metrics of FedDMR under the “w/o Warm” setting show certain improvements compared to other methods, which proves that the dual-layer multi-head attention network and the regularization strategy are effective. Most existing federated recommendation models are based on FedNCF, which often relies solely on the interaction records between users and items for personalized modeling. The structure of these recommendation models is relatively simple. It is difficult for these recommendation models to learn useful information when there are additional user and item attributes available. FedPA introduces low-rank adapters into the recommendation model to model user personalization, achieving improvement compared to other baseline methods. Building on FedPA, FedDMR incorporates the dual-layer multi-head attention network into the recommendation model, further enhancing the understanding of the complex relationships between user embeddings and item embeddings. Meanwhile, FedDMR employs the regularization strategy to guide the updates of local models, ensuring that these updates are constrained by the global model. This further enhances the generalizability of the model. As a result, it achieves significant improvements in AUC and precision compared to FedPA.

**Table 4:** Performance of AUC and precision on three datasets. “w/o Warm” (“w/Warm”) denotes training the federated recommendation system without (with) a pre-trained model. The best results are in bold and the second-best results are underlined. “*” indicate statistically significant improvement (i.e., two-sided *t*-test with p<0.05).

	Method	KuaiRand-Pure		KuaiSAR-S		KuaiSAR-R	
		AUC	Precision	AUC	Precision	AUC	Precision
w/o Warm	FedNCF	68.46	73.33	55.84	**81.82**	70.83	65.45
	PFedNCF	62.99	72.09	55.31	79.44	67.69	63.81
	FedRecon	65.21	61.72	58.56	76.57	66.65	62.68
	PFedRec	59.48	70.80	56.71	79.42	68.32	63.83
	FedPA	68.97	73.35	57.89	81.04	71.30	65.51
	**FedDMR**	70.52 *	75.07 *	60.39 *	81.57	74.37 *	66.83 *
w/Warm	FedNCF	70.07	74.15	60.78	79.03	71.47	66.38
	PFedNCF	62.78	71.75	57.86	79.24	66.82	63.43
	FedRecon	70.02	74.22	55.68	78.54	65.49	61.31
	PFedRec	62.71	71.73	59.74	79.30	67.50	63.81
	FedPA	70.71	75.12	61.99	**86.98**	72.21	66.70
	**FedDMR**	71.93 *	77.15 *	63.31 *	83.81	75.42 *	68.01 *

### 6.4. Effect of the Dual-Layer Multi-Head Attention Network

To examine the effects of dual-layer multi-head attention network, we introduce several variants as follows:

- FedDMR-NoAtt: FedDMR without dual-layer multi-head attention.
- FedDMR-OneAtt: FedDMR with single-layer multi-head attention.
- FedDMR-Qitem: FedDMR employing dual-layer multi-head attention with item embeddings as a query.
- FedDMR-MultiAtt: FedDMR employing dual-layer multi-head attention with different numbers of heads, specifically with 2, 5, 8, and 10 heads.

As shown in Table 5, with the gradual introduction of the multi-head attention mechanism, the performance of the model gradually improves. FedDMR-QItem and FedDMR-MultiAtt incorporate the dual-layer multi-head attention mechanism with different settings; they show certain performance improvements compared to FedDMR-OneAtt, which applies a single-layer multi-head attention mechanism. Compared to the default user-as-query setting, FedDMR-QItem treats item embeddings as the query. This reversal shifts the attention perspective from user-centric to item-centric, impairing the model’s capacity to express users’ individual preferences and leading to observable performance degradation. For FedDMR-MultiAtt, we adjust the number of attention heads to assess the impact of different head counts on model performance. The experimental results indicate that moderately increasing the number of attention heads helps the model to better capture the interactions between users and items. However, excessive heads can degrade model performance, because they may complicate the interactions between users and items, making it difficult for the model to learn effectively. Specifically, we found that 4 attention heads (FedDMR) achieve optimal performance in our experiments, which may be because we chose four attributes for users and items, respectively, from each dataset for modeling embeddings. This design allows each attribute embedding to be independently attended to by a dedicated attention head, thus providing a good balance between model complexity and performance.

To further interpret the effect of the dual-layer multi-head attention network, we conduct a case study on the aggregative attention layer. We compare the recommendation results obtained with (FedDMR) and without (FedDMR-OneAtt) the aggregative attention layer. The results show that the performance improves after introducing the second attention layer. In our dual-layer multi-head attention network, the interactive attention layer mainly focuses on items that users have recently interacted with, reflecting short-term preferences. In contrast, the aggregative attention layer captures semantically related or co-occurring items, effectively integrating higher-order interactions that represent long-term interests.

For example, in the KuaiRand-Pure dataset, user A, who frequently watches videos with the tag attribute “sports”, is selected as a representative case. In the single-layer model, the top recommended videos mainly include those that the user has recently watched, such as basketball and soccer games that reflect the model’s focus on short-term interactions. In contrast, after introducing the second (aggregative) attention layer, the recommended list expands to include videos with tag attributes “comedy” and “game”. This may be because these videos all have high view counts (greater than 10,000); therefore, they share the same complete_play_cnt attribute values. These videos are not directly interacted with by user A but share higher-order correlations with previously watched videos. This indicates that the second layer helps generalize user preferences beyond immediate interactions, improving the diversity and relevance of recommendations.

### 6.5. Analysis of the Regularization Strategy

To examine the effects of each regularizer in the regularization strategy, we introduce three variants as follows:

- FedDMR-NoReg: FedDMR without a regularization strategy.
- FedDMR-Nodiff: FedDMR without a discrepancy regularizer.
- FedDMR-NoL1: FedDMR without an L1 regularizer.

As shown in Table 6, the discrepancy regularizer has a significant impact on model performance, whereas the L1 regularizer has a relatively minor effect. This is because the discrepancy regularizer primarily serves to constrain local models’ updates, ensuring the performance of the global model. Then, the L1 regularizer plays a supplementary role by penalizing the complexity of model parameters. This demonstrates that the regularization strategy we employed is effective in improving the model’s generalizability and preventing overfitting.

**Figure 2:**
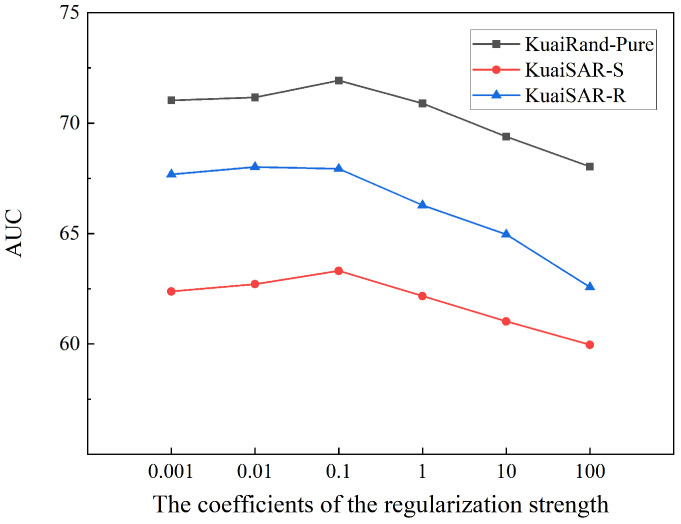
Impact of the regularization strength on model performance.

In addition, we further investigate the sensitivity of FedDMR to the regularization strength coefficients λ and μ. Figure 2 illustrates the trends in AUC as the coefficients of the regularization strength vary on three datasets. We conduct experiments with coefficients in the range of $\left\{10^{i}|i=-3,-2,-1,0,1,2\right\}$. The results show that the model performance remains relatively stable when λ and μ vary from 0.01 to 1, and both excessively small or large values lead to a decline in AUC. Specifically, when the regularization strength is too weak, FedDMR tends to overfit local data, while overly strong constraints restrict personalization and cause underfitting. Optimal performance is achieved when λ and μ are set to 0.1, indicating that moderate regularization strength can balance global generalization and local adaptation. Different datasets respond differently to regularization strength because the characteristics of different datasets (such as data distribution, number of features, etc.) may affect the effectiveness of regularization.

### 6.6. Analysis of Public Parameter Updates

**Figure 3:**
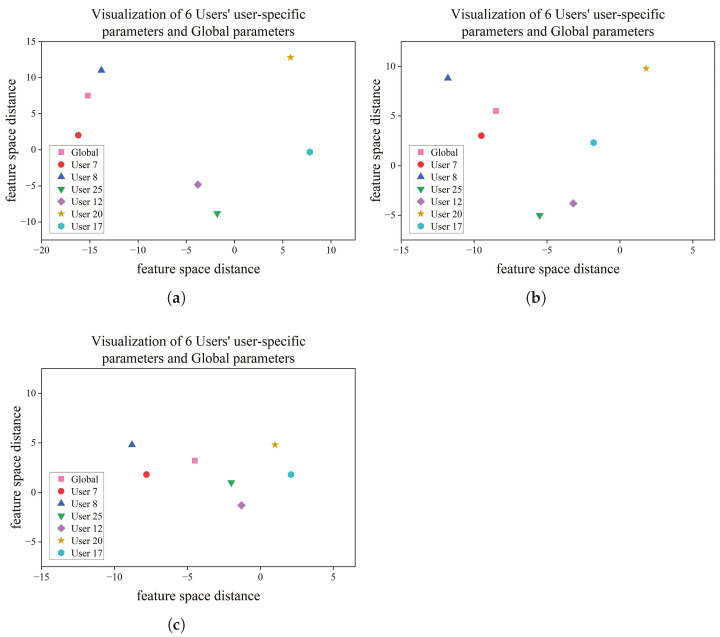
The distribution between user-specific output layer parameters and global output layer parameters in the feature space. (**a**) Round 1. (**b**) Round 20. (**c**) Round 40.

We select representative public parameters, specifically the output layer parameters of the prediction function for the experiment. Figure 3 shows the distribution between the output layer parameters of six randomly selected users and the global aggregated output layer parameters during the 1st, 20th, and 40th training rounds. At the beginning of training, user-specific parameters are relatively dispersed and subject to fewer global constraints. As training progresses, the distribution of user-specific parameters becomes tighter and more consistent with the global parameters in the feature space. This is likely because the regularization strategy effectively limits the excessive deviation in the local models of users, imposing stricter global constraints on the updates of local models and bringing the distributions of user-specific and global output layer parameters closer together. The output layer parameters of different users show both a certain degree of alignment and appropriate distribution differences in the feature space. This means that the model can capture the personalized preferences of local users while maintaining global consistency, thus having better generalizability across different clients.

### 6.7. Analysis of the Model Scalability and Efficiency

Since dual-layer multi-head attention networks are computationally intensive, they pose challenges in deploying this attention network to clients with limited computational resources. To address this issue, we develop a lightweight attention network with the Linformer technique. To examine the effects of the lightweight attention network, we introduce several variants as follows:

- FedDMR w/o att-net: FedDMR without a multi-head attention network, only trains basic recommendation models.
- FedDMR w/ Linatt-net: FedDMR with lightweight multi-head attention networks using the Linformer technique.

As shown in Table 7, training dual-layer multi-head attention networks introduces an average of 10 s of communication costs across three datasets. The Linatt-net, using the Linformer technique to compress the attention matrices into low-rank matrices, achieves an average of 7 s of improvement compared to the original attention networks. Although the training time is still higher compared to the model without using an attention network, it remains within an acceptable range, and its model performance is still considerable.

To validate the performance of lightweight multi-head attention networks using the Linformer technique, we compress the interaction sequence length of users into different low-rank *k*. The interaction sequence length of users in the datasets ranges from 10 to 430, then we set *k* to 10, 8, and 4, respectively. As shown in Table 8, for FedDMR with Linatt-net, the decrease compared to the original attention network is not significant, and it achieves improvements in computational efficiency. The above results strengthen our method’s scalability in real-world applications.

### 6.8. Analysis of the Convergence Trend

**Figure 4:**
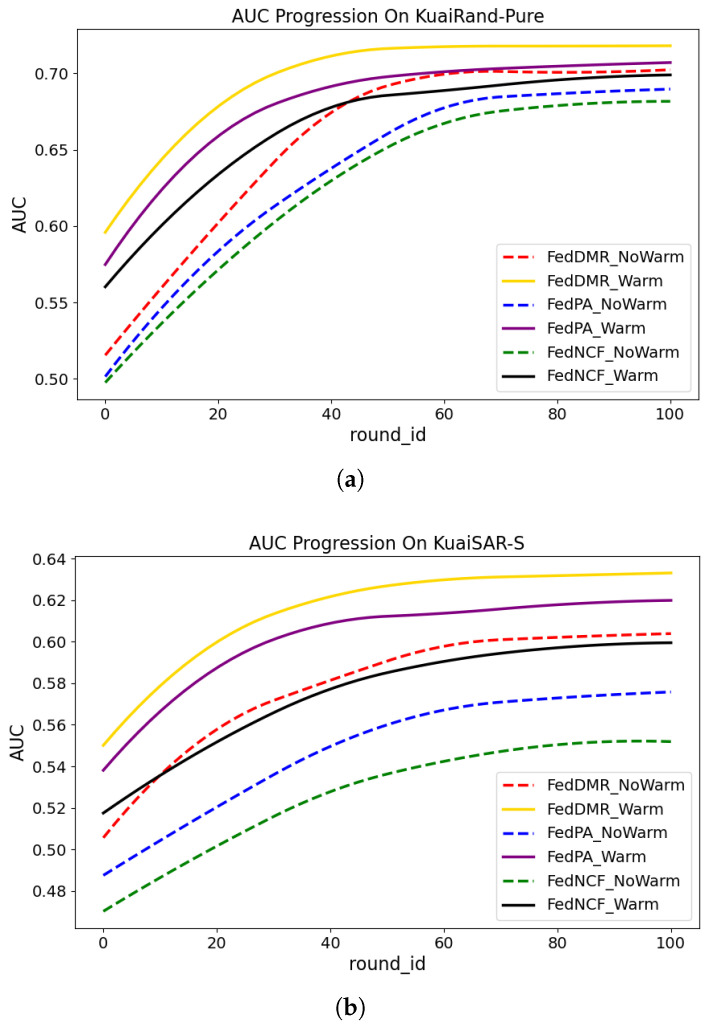
Convergence comparison of different methods on KuaiRand-Pure and KuaiSAR-S. (**a**) KuaiRand-Pure. (**b**) KuaiSAR-S.

Figure 4 shows the changes in AUC with training rounds (round_id) of different methods on two datasets (KuaiRand-Pure and KuaiSAR-S). We compare the following methods:

- FedDMR: The method proposed in this paper.
- FedDMR_NoWarm: A variant of FedDMR without pre-trained models.
- FedPA: One of the baseline methods with pre-trained models.
- FedPA_NoWarm: A variant of FedPA without pre-trained models.
- FedNCF: One of the baseline methods with pre-trained models.
- FedNCF_NoWarm: A variant of FedNCF without pre-trained models.

As can be seen from the results in Figure 4, the same method shows better performance when using pre-trained models for warm-starting, indicating that pre-trained models contribute to enhancing model performance. Methods without pre-trained models converge at around 60 rounds, while on the KuaiRand-Pure dataset, methods using pre-trained models converge at around 40 rounds, and on the KuaiSAR-S dataset, methods using pre-trained models converge at around 50 rounds. This indicates that using pre-trained models can accelerate the convergence speed. Moreover, FedDMR achieves the best performance in all cases. Even without using pre-trained models, its performance is comparable to that of FedNCF after warm-starting, which confirms the effectiveness of the regularization strategy and the dual-layer multi-head attention mechanism.

### 6.9. Protection with Local Differential Privacy

We integrate the local differential privacy (LDP) technique into FedDMR to protect user privacy. Specifically, we add Laplacian noise to the client’s public parameters when uploading them to the server. We set the noise intensity from 0.1 to 0.5 to observe the effects. The experimental results in Table 9 show that performance declines slightly as the noise strength grows. However, when noise is not too strong, the decline is minimal. Therefore, an appropriate noise intensity is required to balance model performance and privacy protection.

## 7. Conclusions

This paper proposes a novel federated recommendation framework called FedDMR, which integrates pre-trained models with a dual-layer multi-head attention network and a regularization strategy. FedDMR designs the dual-layer multi-head attention network to capture the complex interactions between user and item embeddings, which enables the model to more comprehensively understand users’ personalized preferences. It addresses the challenges of overfitting using regularizers to constrain local model updates, ensuring the consistency of the local model’s public parameters with global parameters during the update process. Experiments on three benchmark datasets demonstrate that FedDMR outperforms state-of-the-art federated recommendation methods, achieving significant improvements in AUC and precision. The study also includes an analysis of the impact of regularization strength and the effectiveness of the multi-head attention network, and provides insights into balancing model performance and privacy protection in federated recommendation systems. However, our method cannot simulate changes in user preferences or the appearance of new items. As our model is training on the offline static datasets, the model needs to be retrained to adapt to new data when users’ preferences change or new items are introduced. We recognize this as a potential limitation of the model and consider the model’s ability for incremental learning and online learning as an important direction for future work.

## Figures and Tables

**Table 1:** Main notations.

Notations	Description
$u_{emb}^{i}$	Embedding vector of user *i*
$v_{emb}^{j}$	Embedding vector of item *j*
$E_{u}$, $E_{v}$	User and item embedding tables
$r_{ij}$	True label of user–item interactions (1 if interacted, 0 otherwise)
${\hat{r}}_{ij}$	Predicted rating for user *i* on item *j*
$\theta_{rec}^{i}$	Basic recommendation model parameters of user *i*
$\theta_{ue}$	User embedding table parameters
$\theta_{ve}$	Item embedding table parameters
$\theta_{mlp}^{i}$	MLP parameters of user *i*
$int_{emb}^{ij}$	Interactive attention embeddings for user *i* on item *j*
$att_{emb}^{ij}$	Aggregative attention embeddings for user *i* on item *j*
*H*	The number of attention heads
$W_{h}^{Q}$, $W_{h}^{K}$, $W_{h}^{V}$	The weight matrix for the *h* attention head
$\theta_{\mathrm{public}\;}^{i}$	Public shared parameters for client(user) *i*
$\theta_{\mathrm{global}\;}$	Global parameters
λ, μ	The coefficients of the regularization strength

**Table 2:** Basic statistics of the datasets.

Dataset	Users	Items	Interactions
KuaiRand-Pure	27,285	7551	1,436,609
KuaiSAR-S	25,877	2,012,476	3,171,231
KuaiSAR-R	25,877	2,281,034	7,493,101

**Table 3:** Hyperparameter settings.

Hyperparameter	Value/Setting
Learning rate	0.1
Batch size	1024
Embedding dimension	8
MLP hidden layers	2
Optimizer	Adam
Weight decay	$1\times 10^{-5}$
Regularization coefficients	$\left\{10^{i}|i=-3,-2,-1,0,1,2\right\}$
Parameter initialization	Pre-trained weights/Default random initialization
Random seed	0

**Table 5:** Ablation study of dual-layer multi-head attention on the KuaiRand-Pure dataset.

Method		Metric	
		AUC	Precision
FedDMR-NoAtt		70.82	75.33
FedDMR-OneAtt		71.33	75.89
FedDMR-QItem		71.43	76.06
FedDMR-MultiAtt	head = 2	71.46	75.86
	head = 5	71.61	76.27
	head = 8	71.32	76.11
	head = 10	71.03	75.77
FedDMR		71.93	77.15

**Table 6:** Ablation study of the regularization strategy on KuaiRand-Pure.

Method	Metric	
	AUC	Precision
FedDMR-NoReg	71.62	76.79
FedDMR-Nodiff	71.67	76.77
FedDMR-NoL1	71.85	77.13
FedDMR	71.93	77.15

**Table 7:** Training time for each round of different variants in attention networks.

Methods	Datasets		
	KuaiRand-Pure	KuaiSAR-S	KuaiSAR-R
FedDMR w/o att-net	108.31 s	117.18 s	124.89 s
FedDMR	120.82 s	130.04 s	140.21 s
FedDMR w/Linatt-net	112.52 s	123.77 s	130.37 s

**Table 8:** Experimental results of recommendation models with different attention networks on KuaiRand-Pure.

Methods		Metric	
		AUC	Precision
FedDMR w/o att-net		70.82	75.33
FedDMR		71.93	77.15
FedDMR w/Linatt-net	k = 10	71.85	77.12
	k = 8	71.77	76.89
	k = 4	71.52	76.53

**Table 9:** Performance of FedDMR with various noise strengths.

Dataset	Noise Strength	0	0.1	0.2	0.3	0.4	0.5
KuaiRand-Pure	AUC	71.93	71.79	71.67	71.57	71.49	71.45
	Precision	77.15	77.03	76.97	76.91	76.74	76.59

## Data Availability

The data presented in this study are available on request from the corresponding author.

## References

[1] Chai D., Wang L., Chen K., Yang Q. (2020). Secure federated matrix factorization. IEEE Intell. Syst..

[2] Javeed D., Saeed M.S., Kumar P., Jolfaei A., Islam S., Islam A.N. (2023). Federated learning-based personalized recommendation systems: An overview on security and privacy challenges. IEEE Trans. Consum. Electron..

[3] McMahan B., Moore E., Ramage D., Hampson S., y Arcas B.A. Communication-efficient learning of deep networks from decentralized data. Proceedings of the Artificial Intelligence and Statistics.

[4] Ammad-Ud-Din M., Ivannikova E., Khan S.A., Oyomno W., Fu Q., Tan K.E., Flanagan A. (2019). Federated collaborative filtering for privacy-preserving personalized recommendation system. arXiv.

[5] Perifanis V., Efraimidis P.S. (2022). Federated neural collaborative filtering. Knowl.-Based Syst..

[6] Zhang C., Long G., Zhou T., Yan P., Zhang Z., Zhang C., Yang B. (2023). Dual personalization on federated recommendation. arXiv.

[7] Lin Y., Ren P., Chen Z., Ren Z., Yu D., Ma J., Rijke M.d., Cheng X. Meta matrix factorization for federated rating predictions. Proceedings of the 43rd International ACM SIGIR Conference on Research and Development in Information Retrieval.

[8] Li Z., Long G., Zhou T. (2023). Federated recommendation with additive personalization. arXiv.

[9] Zhang C., Long G., Guo H., Fang X., Song Y., Liu Z., Zhou G., Zhang Z., Liu Y., Yang B. (2024). Federated adaptation for foundation model-based recommendations. arXiv.

[10] He X., Liao L., Zhang H., Nie L., Hu X., Chua T.S. Neural collaborative filtering. Proceedings of the 26th International Conference on World Wide Web.

[11] Vaswani A., Shazeer N., Parmar N., Uszkoreit J., Jones L., Gomez A.N., Kaiser Ł., Polosukhin I. Attention is all you need. Proceedings of the 31st Conference on Neural Information Processing Systems (NIPS 2017).

[12] Wei S., Meng S., Li Q., Zhou X., Qi L., Xu X. (2023). Edge-enabled federated sequential recommendation with knowledge-aware Transformer. Future Gener. Comput. Syst..

[13] Belhadi A., Djenouri Y., de Alcantara Andrade F.A., Srivastava G. (2024). Federated constrastive learning and visual transformers for personal recommendation. Cogn. Comput..

[14] Feng C., Feng D., Huang G., Liu Z., Wang Z., Xia X.G. (2024). Robust privacy-preserving recommendation systems driven by multimodal federated learning. IEEE Trans. Neural Netw. Learn. Syst..

[15] Li L., Zhang Z., Huang C., Zhang J. (2024). Semi-global sequential recommendation via EM-like federated training. Expert Syst. Appl..

[16] Zhang C., Long G., Guo H., Liu Z., Zhou G., Zhang Z., Liu Y., Yang B. Multifaceted user modeling in recommendation: A federated foundation models approach. Proceedings of the AAAI Conference on Artificial Intelligence.

[17] Dialameh M., Hamzeh A., Rahmani H., Dialameh S., Kwon H.J. (2024). DL-Reg: A deep learning regularization technique using linear regression. Expert Syst. Appl..

[18] Srivastava N., Hinton G., Krizhevsky A., Sutskever I., Salakhutdinov R. (2014). Dropout: A simple way to prevent neural networks from overfitting. J. Mach. Learn. Res..

[19] Laskin M., Lee K., Stooke A., Pinto L., Abbeel P., Srinivas A. (2020). Reinforcement learning with augmented data. Adv. Neural Inf. Process. Syst..

[20] Li Z., Long G., Zhang C., Zhang H., Jiang J., Zhang C. (2024). Navigating the future of federated recommendation systems with foundation models. arXiv.

[21] Zhang H., Liu H., Li H., Li Y. (2024). Transfr: Transferable federated recommendation with pre-trained language models. arXiv.

[22] Muhammad K., Wang Q., O’Reilly-Morgan D., Tragos E., Smyth B., Hurley N., Geraci J., Lawlor A. Fedfast: Going beyond average for faster training of federated recommender systems. Proceedings of the 26th ACM SIGKDD International Conference on Knowledge Discovery & Data Mining.

[23] Pillutla K., Malik K., Mohamed A.R., Rabbat M., Sanjabi M., Xiao L. Federated learning with partial model personalization. Proceedings of the International Conference on Machine Learning.

[24] Wu C., Wu F., Cao Y., Huang Y., Xie X. (2021). Fedgnn: Federated graph neural network for privacy-preserving recommendation. arXiv.

[25] Singhal K., Sidahmed H., Garrett Z., Wu S., Rush J., Prakash S. (2021). Federated reconstruction: Partially local federated learning. Adv. Neural Inf. Process. Syst..

[26] Yu E., Ye Z., Zhang Z., Qian L., Xie M. (2024). A federated recommendation algorithm based on user clustering and meta-learning. Appl. Soft Comput..

[27] Li Z., Long G., Zhou T., Jiang J., Zhang C. Personalized federated collaborative filtering: A variational autoencoder approach. Proceedings of the AAAI Conference on Artificial Intelligence.

[28] Zhang C., Long G., Zhou T., Zhang Z., Yan P., Yang B. When federated recommendation meets cold-start problem: Separating item attributes and user interactions. Proceedings of the ACM Web Conference 2024.

[29] Huynh T.T., Nguyen T.B., Nguyen T.T., Nguyen P.L., Yin H., Nguyen Q.V.H., Nguyen T.T. (2025). Certified unlearning for federated recommendation. ACM Trans. Inf. Syst..

[30] Han M., Li D., Xia J., Liu J., Gu H., Zhang P., Gu N., Lu T. FedCIA: Federated Collaborative Information Aggregation for Privacy-Preserving Recommendation. Proceedings of the 48th International ACM SIGIR Conference on Research and Development in Information Retrieval.

[31] Zhou G., Zhu X., Song C., Fan Y., Zhu H., Ma X., Yan Y., Jin J., Li H., Gai K. Deep interest network for click-through rate prediction. Proceedings of the 24th ACM SIGKDD International Conference on Knowledge Discovery & Data Mining.

[32] Kang W.C., McAuley J. (2018). Self-attentive sequential recommendation. Proceedings of the 2018 IEEE International Conference on Data Mining (ICDM).

[33] Sun F., Liu J., Wu J., Pei C., Lin X., Ou W., Jiang P. BERT4Rec: Sequential recommendation with bidirectional encoder representations from transformer. Proceedings of the 28th ACM International Conference on Information and Knowledge Management.

[34] Du Y., Liu H., Wu Z. (2021). Modeling multi-factor and multi-faceted preferences over sequential networks for next item recommendation. Proceedings of the Joint European Conference on Machine Learning and Knowledge Discovery in Databases.

[35] Xiao C., Xie R., Yao Y., Liu Z., Sun M., Zhang X., Lin L. (2021). Uprec: User-aware pre-training for recommender systems. arXiv.

[36] Zhao Y., Li M., Lai L., Suda N., Civin D., Chandra V. (2018). Federated learning with non-iid data. arXiv.

[37] Li T., Sahu A.K., Zaheer M., Sanjabi M., Talwalkar A., Smith V. (2020). Federated optimization in heterogeneous networks. Proc. Mach. Learn. Syst..

[38] Acar D.A.E., Zhao Y., Navarro R.M., Mattina M., Whatmough P.N., Saligrama V. (2021). Federated learning based on dynamic regularization. arXiv.

[39] Xu C., Hong Z., Huang M., Jiang T. (2022). Acceleration of federated learning with alleviated forgetting in local training. arXiv.

[40] Ko H., Lee S., Park Y., Choi A. (2022). A survey of recommendation systems: Recommendation models, techniques, and application fields. Electronics.

[41] Zhang C., Xie Y., Bai H., Yu B., Li W., Gao Y. (2021). A survey on federated learning. Knowl.-Based Syst..

[42] Choi W.S., Tomei M., Vicarte J.R.S., Hanumolu P.K., Kumar R. (2018). Guaranteeing local differential privacy on ultra-low-power systems. Proceedings of the 2018 ACM/IEEE 45th Annual International Symposium on Computer Architecture (ISCA).

[43] Wang S., Li B.Z., Khabsa M., Fang H., Ma H. (2006). Linformer: Self-attention with linear complexity (2020). arXiv.

[44] Gao C., Li S., Zhang Y., Chen J., Li B., Lei W., Jiang P., He X. Kuairand: An unbiased sequential recommendation dataset with randomly exposed videos. Proceedings of the 31st ACM International Conference on Information & Knowledge Management.

[45] Sun Z., Si Z., Zang X., Leng D., Niu Y., Song Y., Zhang X., Xu J. KuaiSar: A unified search and recommendation dataset. Proceedings of the 32nd ACM International Conference on Information and Knowledge Management.

